# Nausea Following Anæsthesia

**Published:** 1892-06

**Authors:** 


### Nausea Following Anesthesia.

If the patient has a great deal of nausea after etherization, what can you do? The books generally dismiss it by saying it will pass off. When you have such a case, you will feel like passing off yourself. Iced drinks and carbonic acid water are good. One of the best remedies is chloroform, four or five drops, with two or three drops of vinegar of opium, given two or three times a day. That will sometimes allay vomiting. Another plan, when you have reason to think there will be great nausea or vomiting, is to put your patient to sleep. A great many surgeons are opposed to morphine or opium after operation. Before the operation I am apt to give a little brandy or whisky, and a little morphine hypodermically; in that way I do away with the necessity of giving a large amount of ether. Usually, after operation, I order a hypodermic—one-sixth grain of morphine. It is not only to alleviate the pain, but to quiet the patient and the stomach. It controls the nausea and puts the patient to sleep, giving the stomach and nervous system time to recover themselves.—*Brinton.*

To Dissolve Cocaine.—Squibb recommends the use of a half of 1 per cent. solution of boric acid to dissolve cocaine, this amount being needed to prevent decomposition.

